# Enhanced Toughness and Ductility of Friction Stir Welded SA516 Gr.70 Steel Joint via Post-Welding Annealing

**DOI:** 10.3390/ma17010116

**Published:** 2023-12-25

**Authors:** Xiuying Wang, Ziqi Miao, Wenbiao Gong, Guipeng Lu, Juncai Sun, Yuqian Wang, Guangming Xie

**Affiliations:** 1 Institute of Materials and Technology, Dalian Maritime University, Dalian 116026, China; janewxy@cpm.com.cn; 2 The Challenge Petrochemical Machinery Corporation of Maoming (CPM), Maoming 525024, China; 3 School of Materials Science and Engineering, Changchun University of Technology, Changchun 130012, China; 13756790688@163.com (Z.M.); gwbiao@163.com (W.G.); 4 FAW Tooling Die Manufacturing Co., Ltd., Changchun 130013, China; neuwangc@163.com; 5 State Key Laboratory of Rolling and Automation, Northeastern University, No. 3 Wenhua Road, Shenyang 110819, China; yqwangneu@126.com

**Keywords:** friction stir welding, shielded metal arc welding, SA516 Gr.70 steel, annealing, toughness and ductility

## Abstract

The SA516 Gr.70 steel possessing excellent toughness and plasticity has been widely used in the cryogenic field. However, the appearance of coarse bainite in the heat affected zone (HAZ) of the fusion welded joint deteriorates the toughness and ductility. In this work, 4.5 mm thick SA516 Gr.70 steel was joined using shielded metal arc welding (SMAW) and friction stir welding (FSW), respectively, and the microstructure and mechanical properties of joints were investigated in detail. The Charpy energy in the HAZ in the FSW joint was 80 J/cm^2^, which was higher than that of the HAZ in the SMAW joint (60 J/cm^2^) and due to microstructure refinement. In addition, the total elongation (TE) of the SMAW joint was 17.5%, which was higher than that of the FSW joint (12.1%) and caused by a wider nugget zone with high hardness. The post-welding annealing was used to improve the toughness and ductility of the SMAW and FSW joints, and the microstructure and mechanical properties of the joints after annealing were analyzed. The toughness in the HAZ of the SMAW and FSW joints were 80 and 103 J/cm^2^, and the TE of the SMAW and FSW joints were 18.6% and 25.2%, respectively. Finally, the as-annealed FSW joints exhibited excellent toughness and ductility. The abovementioned excellent mechanical properties were primarily attributed to the appearance of tempering martensite, decrease in dislocation density, and fine grain.

## 1. Introduction

The normalized microstructure of SA516 Gr.70 steel is typical pearlite and ferrite with excellent toughness and plasticity, which is widely used in large structures such as pressure vessels, transportation pipelines, ships, and bridges [1,2]. At present, SA516 Gr. 70 steel is mainly welded by fusion welding technologies, and welded joints are a key part of large structures, often becoming the weakest area [3,4,5,6]. Under the high heat input of fusion welding, the presence of coarse grain and brittle phases seriously deteriorates the toughness and plasticity of the joint, especially in the heat affected zone (HAZ) [4]. For example, Teske et al. [5] carried out gas metal arc welding (GMAW) on SA516 Gr.70 steel and found that the impact energy of the joint was only 19 J, which was lower than the minimum value (20 J) required by standard ASTM A20/A20M-89a for ASTM A516 grade 70 steel due to the formation of a coarse microstructure [7]. In addition, Pritesh et al. [8] performed GMAW on 10 mm thick SA516Gr70 steel plates and pointed out that the elongation of the welded joint was 41% of that of the base metal (BM).

As a solid-phase joining technology, friction stir welding (FSW) is widely used to join aluminum alloys, achieving good application results [9]. In recent years, FSW research has also begun to shift from non-ferrous metals to ferrous metals with high melting points, realizing reliable connections of pipeline steel, DP steel, and TRIP steel [10,11,12]. Compared with traditional fusion welding, the HAZ of FSW steel joint is narrow and the coarsening and embrittlement of microstructures in the joint are not obvious. In addition, there is no obvious element segregation, porosity, crack, and other defects in FSW joints, thereby producing excellent toughness and ductility due to the low heat input characteristics, which has great potential in the field of advanced steel connection [13]. Previously, Duan et al. [14] carried out double-sided FSW and gas metal arc welding (GMAW) on 11 mm thick X80 pipeline steel plates, respectively, and systematically studied the relationship between microstructure and toughness in the joints. It was found that the HAZ of the GMAW joint was mainly composed of coarse granular bainite (GB) and coarse network-like martensite-austenite (M–A) constituents, while the GB in the HAZ of the FSW joints was refined, and the network-like M–A constituent was absent. As a result, the impact energy in the HAZ of the FSW joint was 89% of that of the BM, which was much higher than that of the GMAW joint. At the same time, Wang et al. [15] performed tungsten inert gas (TIG) welding and FSW on quenching and partitioning 1180 steel and found that the microstructure in TIG welding joint consisted of coarse martensite, while the martensite in FSW joint was clearly refined due to strong plastic deformation and low heat input. Therefore, excellent tensile strength, as high as that of the BM, was obtained in the FSW joints, whereas dramatic losses of strength and plasticity were found in the TIG welded joint. In addition, Qiao et al. [16] carried out FSW on high Mn twin-induced plasticity steel and found that no element volatilization and segregation was found in the joint, thus improving the strength and plasticity. Accordingly, when FSW was applied to joining SA516 Gr.70 steel, this technology could inhibit microstructural coarsening, improving the mechanical properties. Lim et al. [17] fabricated a thick multilayered A516 Grade 70 steel structure by multipass FSW, exhibiting good toughness and ductility depending on microstructure refinement. Therefore, FSW is expected to improve the toughness and plasticity of the SA516 Gr.70 steel welded joint. However, studies on the microstructure evolution and mechanical properties of FSW SA516 Gr.70 steel joints have yet to be reported.

In this work, the SA516 Gr.70 steel was joined via FSW, and post-welding annealing was performed on the SA516 Gr.70 steel joints to enhance the plasticity and toughness. The results of the shielded metal arc welding (SMAW) are also compared with the FSW process. In addition, the microstructure and mechanical properties of the as-welded and as-annealed SA516 Gr.70 steel joints were systematically investigated.

## 2. Experimental Procedure

In this work, a 4.5 mm thick hot-rolled SA516 Gr.70 steel was normalized at 800 °C for 2 h, which was used as a basal metal (BM). The chemical composition of SA516 Gr.70 steel is shown in Table 1. The SA516 Gr.70 steel plates are joined using FSW and SMAW. FSW was performed at a rotation rate of 400 rpm and a traverse speed of 100 mm/min. FSW and SMAW equipment were manufactured by Beijing FSW Technology Co., Ltd., (Beijing, China) and Andeli Group Co., Ltd., (Zhejiang, China). A W-25Re (wt.%) tool that consisted of a 12 mm diameter concave shoulder, a root diameter of 8 mm, and a length of 4 mm tapered thread stirring pin was used. During FSW, the tilt angle of the tool was maintained at 3° from the normal direction of the plate. Before SMAW, the SA516 Gr.70 steel was cleaned, and V grooves (60°) were machined. During the SMAW process, the welding direction was parallel to the rolling direction, an arc voltage of 20–22 V, a welding current of 90–120 A, a welding speed of 15 cm·min^−1^, and filler metal of E7018-1H4R were utilized. After SMAW and FSW, the joint was annealed at 620 °C for 2 h and then cooled to the ambient temperature in air.

The microstructure was characterized using an optical microscope (OM, Leica, Germany), scanning electron microscopy (SEM, Zeiss Ultra-55, Jena, Germany), and electron backscatter diffraction (EBSD, Oxford Instruments, Abingdon-on-Thames, UK). The EBSD specimen was electro-polished with a 90% ethanol solution and 10% perchloric acid at 20 V for 20 s. To characterize the element distribution in the SMAW joint, specimens were conducted in an electron probe microanalyzer (EPMA, JXA-8530F, JEOL Co., Ltd., Kyoto, Japan) at 20 kV and 2 × 10^−8^ A. The EPMA specimens were mechanically polished and etched with 4% nital.

**Figure 1:**
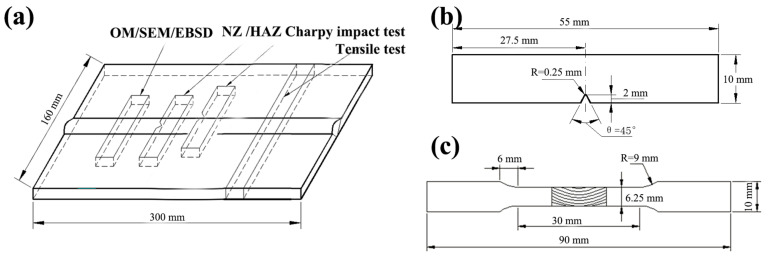
Schematic diagram of sample locations (**a**), Charpy V-notch sample dimension (**b**), tension sample dimension of welded joint (**c**).

The Vickers hardness of the cross-sectional joint was evaluated with a load of 100 g and a dwell time of 10 s using a Future-Tech FM-700 micro-hardness machine (Kawasaki, Japan). To evaluate the tensile properties of welded joints, the tensile test was carried out with a gauge distance of 25 mm, and the displacement of the gauge section was measured using a video extensometer. The tensile properties of the transverse joint were tested at a strain rate of 1 × 10^−3^ s^−1^ by a tensile machine (GOTECH, AI-7000-LAU10, Taiwan, China). The Charpy V-notch impact toughness of the nugget zone (NZ) and HAZ was tested by an Instron machine at −46 °C (Norwood, MA, USA). The non-standard specimen with a thickness of 2 mm was used to evaluate the Charpy energy of HAZ and NZ of the joint because the thickness of the experimental plates was less than that of the standard impact specimen (10 mm). The tensile and Charpy specimens were machined from the welded plates along the transverse direction (Figure 1a). The dimensions of the Charpy and tensile samples are shown in Figure 1b,c.

## 3. Results and Discussion

### 3.1. Microstructure and Mechanical Properties of the As-Welded SMAW and FSW Joints

**Figure 2:**
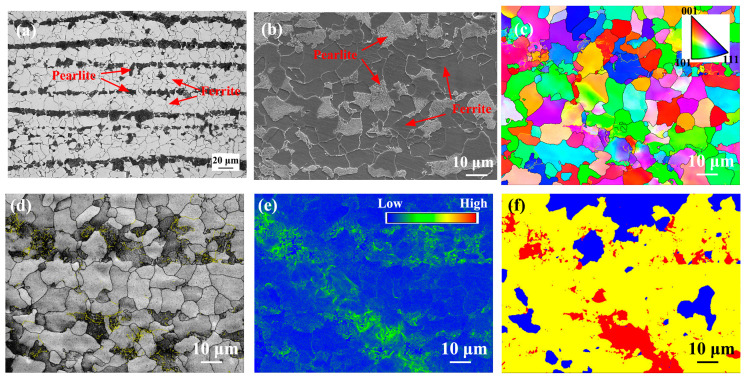
Microstructure images of the BM. (**a**) OM, (**b**) SEM, (**c**) EBSD-inverse pole figure (IPF), (**d**) grain boundary distribution map, (**e**) kernel average misorientation (KAM) map, and (**f**) recrystallization map.

Figure 2 shows microstructure images of the BM. The BM is composed of typical pearlite and polygonal ferrite, and the polygonal ferrite and pearlite are distributed alternately perpendicular to the rolling direction (Figure 2a,b). Figure 2c–f shows EBSD images of the BM. In the grain boundary distribution map, the black and yellow lines represent the high-angle boundaries (HABs, misorientation angle ≥ 15°) and low-angle boundaries (LABs, 2° ≤ misorientation angle < 15°), respectively. It can be found that the grains in the BM are mainly equiaxed and without obvious orientation distribution (Figure 2c). In addition, the BM exhibited a low kernel average misorientation (KAM) value. The recrystallization distribution map is used to characterize the distribution of deformation microstructure, substructure microstructure, and recrystallization microstructure. In Figure 2f, the yellow, red, and blue areas represent substructure, deformation, and recrystallization microstructures, respectively. It is found that the BM mainly contains substructure and recrystallization microstructure and a small amount of deformed microstructure. This should be due to the occurrences of recovery and recrystallization during normalizing after hot rolling, which reduces the amount of deformed microstructure.

**Figure 3:**
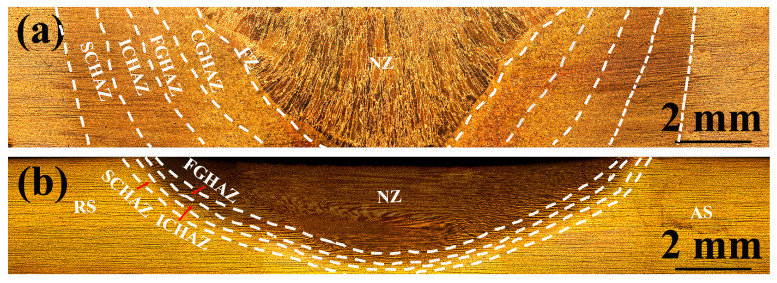
Macrographs of the as-welded SMAW and FSW joints. (**a**) SMAW, (**b**) FSW.

Figure 3 shows macrographs of the as-welded SMAW and FSW SA516 Gr.70 steel joints. There were no visible defects in the as-welded SMAW and FSW joints. The NZ of the FSW joint presented a “basin” shape, where the left side was the forward side (AS) and the right side was the backward side (RS). The welded joints are mainly composed of NZ, fusion zone (FZ), heat affected zone (HAZ), and BM. According to the different peak temperatures, the HAZ can be divided into four sub-zones, which are subcritical HAZ (SCHAZ), intercritical HAZ (ICHAZ), fine-grained HAZ (FGHAZ), and coarse-grained HAZ (CGHAZ), whose peak temperatures approximately are <A_c1_, A_c1_~A_c3_, >A_c3_ and >>A_c3_, respectively [18]. Compared with the SMAW joint, the narrower width of the HAZ in the FSW joints was ascribed to the fact that FSW was solid-phase welding with low heat input. In addition, the FSW joint exhibited a wider NZ relative to the SMAW joint depending on a wider shoulder and stirring needle.

**Figure 4:**
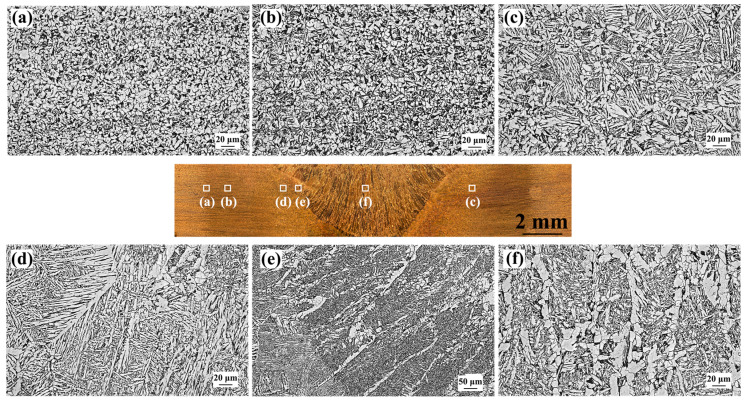
OM images of as-welded SMAW joint (**a**) SCHAZ, (**b**) ICHAZ, (**c**) FGHAZ, (**d**) CGHAZ, (**e**) FZ, (**f**) NZ.

Figure 4 shows OM images of the SMAW SA516 Gr.70 steel joint. The microstructure of SCHAZ contained ferrite and pearlite (Figure 4a). Compared with BM, the amount of pearlite in the SCHAZ obviously decreased. In the ICHAZ, FGHAZ, and CGHAZ, ferrite, lath bainite, and pearlite were obtained. With the increase in peak temperature, the number of ferrite and pearlite decreases, while the amount of bainite increases. The pearlite and ferrite in the original BM gradually transformed into austenite at the peak temperature above A_c1_, and subsequently, the austenite transformed into lath bainite during post-welding cooling due to the rapid cooling rate. Compared with ICHAZ and FGHAZ, the bainite lath in the CGHAZ is longer owing to the coarsening prior austenite caused by the high peak temperature and long residence time at high temperatures during welding. Ultimately, the microstructures of the FZ and NZ were mainly composed of ferrite and bainite. Compared with FZ, the content of ferrites in the NZ increased, which was attributed to the fact that the high peak temperature and slow cooling rate promoted the formation of ferrites.

**Figure 5:**
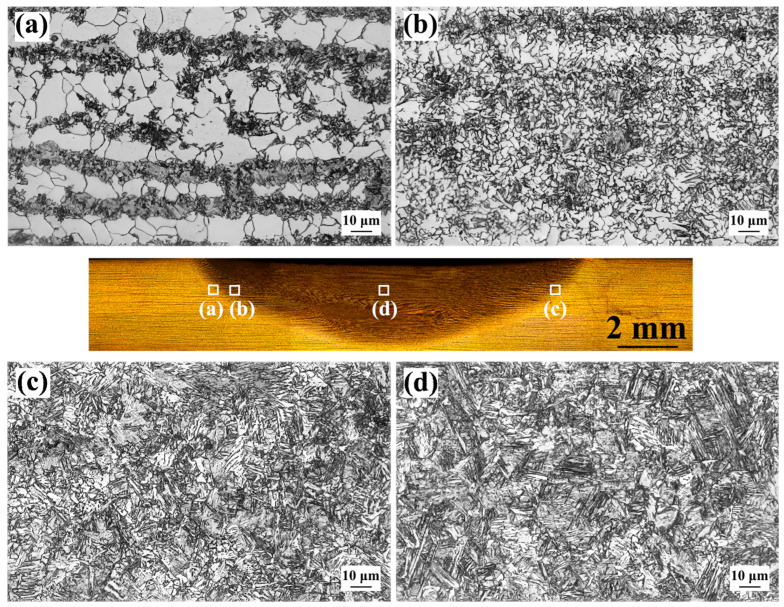
OM images of as-welded FSW joint (**a**) SCHAZ, (**b**) ICHAZ, (**c**) FGHAZ, (**d**) NZ.

Figure 5 shows OM images of the as-welded FSW joint. Unlike the SMAW joint, the FSW joint consisted of BM, HAZ, and NZ, and the HAZ of the FSW joint was divided into SCHAZ, ICHAZ, and FGHAZ; the CGHAZ was not found due to the low heat input of FSW. The microstructure of SCHAZ was ferrite and pearlite, which was similar to that of the BM (Figure 5a). The microstructures of ICHAZ were mainly composed of ferrite, lath bainite, and pearlite (Figure 5b). At the peak temperature of A_c1_~A_c3_, part of pearlite and ferrite in the original BM undergo austenite transformation. In this case, the austenite transforms into bainite due to the high cooling rate during the post-weld cooling. In the FGHAZ and NZ, a large amount of bainite was observed. Compared with SMAW, the microstructure in the HAZ of FSW joints was refined since FSW had low heat input, which suppressed grain growth.

**Figure 6:**
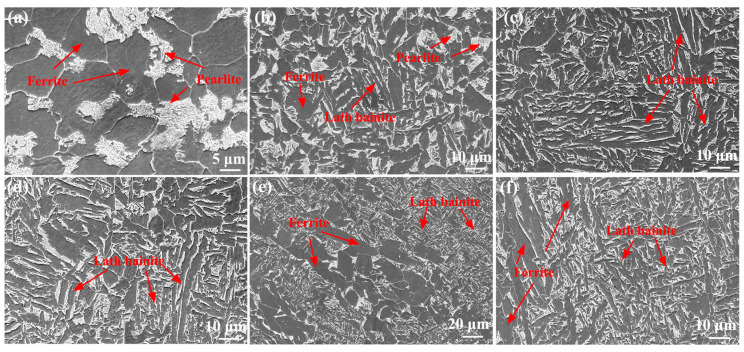
SEM images of as-welded SMAW joint (**a**) SCHAZ, (**b**) ICHAZ, (**c**) FGHAZ, (**d**) CGHAZ, (**e**) FZ, (**f**) NZ.

Figure 6 shows SEM images of the as-welded SMAW joint. The microstructure of the SCHAZ was mainly composed of ferrite and pearlite. The ICHAZ contained ferrite, pearlite, and lath bainite, while the microstructure of the FGHAZ and CGHAZ mainly consisted of lath bainite. Compared with FGHAZ, lath bainite in the CGHAZ was further coarsened. In the FZ and NZ, some ferrite and coarse bainite were obtained, which was attributed to the fact that the peak temperature of FZ and NZ was the highest relative to the HAZ, and the cooling rate post-welding was slower, which promoted the formation of ferrite.

**Figure 7:**
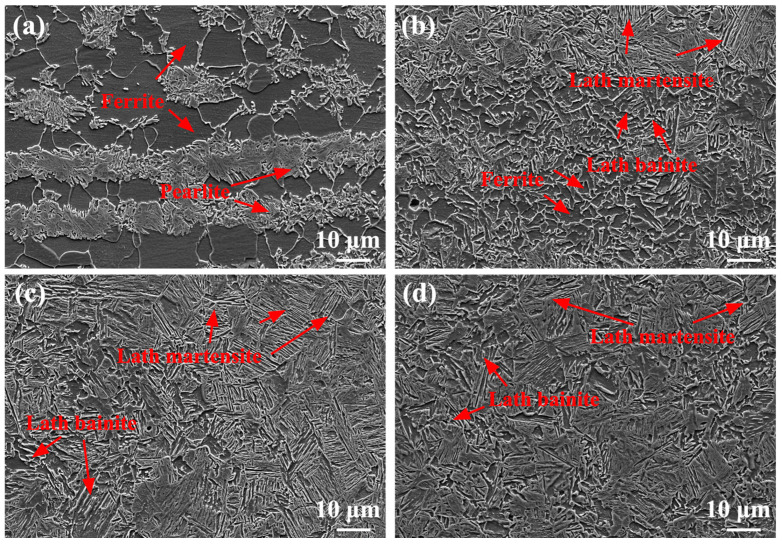
SEM images of as-welded FSW joint (**a**) SCHAZ, (**b**) ICHAZ, (**c**) FGHAZ, (**d**) NZ.

Figure 7 shows SEM images of the as-welded FSW joint. The microstructure of the SCHAZ contained pearlite and ferrite (Figure 7a). In the ICHAZ, some lath martensite is also observed in addition to bainite, pearlite, and ferrite (Figure 7b). For FGHAZ and NZ, lath martensite was also found in addition to lath bainite (Figure 7c). Compared with ICHAZ, the content and size of martensite in the FGHAZ and NZ increased. During FSW process, a low peak temperature and fast post-welding cooling rate promoted the formation of lath martensite. In addition, the peak temperature of the FGHAZ and NZ increased relative to ICHAZ, which resulted in the formation of more austenite at high temperatures, thus increasing the content of martensite and bainite. Compared with SMAW joints, the microstructure of FSW joints was obviously refined, especially FGHAZ and NZ since FSW belonged to solid-phase welding technology, which had low heat input, inhibiting grain growth.

**Figure 8:**
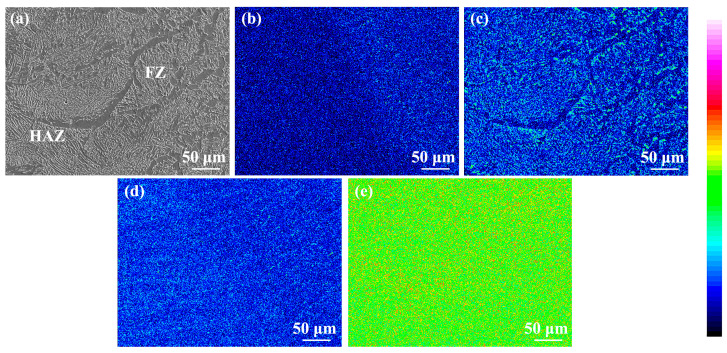
Element distribution maps in the as-welded SMAW joint (**a**) SEM, (**b**) Si, (**c**) C, (**d**) Mn, (**e**) Fe.

Figure 8 shows the element distribution maps in the as-welded SMAW joint. It can be found that there was a clear boundary between the NZ and the HAZ, and the Si content in the NZ was significantly higher than that in the HAZ. The C atoms were dispersed in the NZ and HAZ, while it was mainly concentrated in carbide, and the content of C atoms in ferrite was less. The Mn and Fe elements were uniformly distributed in the joint.

**Figure 9:**
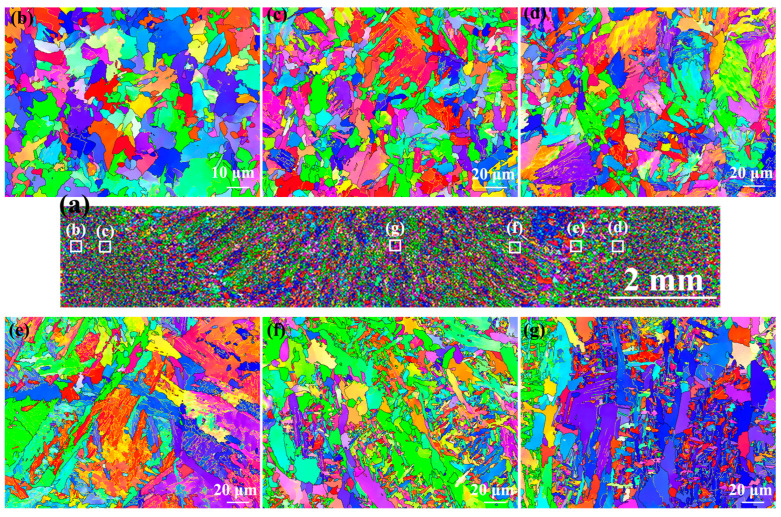
EBSD-IPF maps of the as-welded SMAW joint (**a**) Macroscopic joint, (**b**) SCHAZ, (**c**) ICHAZ, (**d**) FGHAZ, (**e**) CGHAZ, (**f**) FZ, (**g**) NZ.

Figure 9 shows the EBSD-IPF maps of the as-welded SMAW joint. It can be observed that the microstructure of the NZ exhibited cast-like morphology, which was significantly different from the HAZ (Figure 9a). In addition, there was no obvious orientation distribution of the grains in the various regions of the welded joint. In the HAZ, the grain size increased markedly with decreasing distance from the center of the NZ depending on the increase in peak temperature (Figure 9b–g). However, with respect to the FZ and NZ, the average grain size decreased, which corresponded to the presence of fine ferrites.

**Figure 10:**
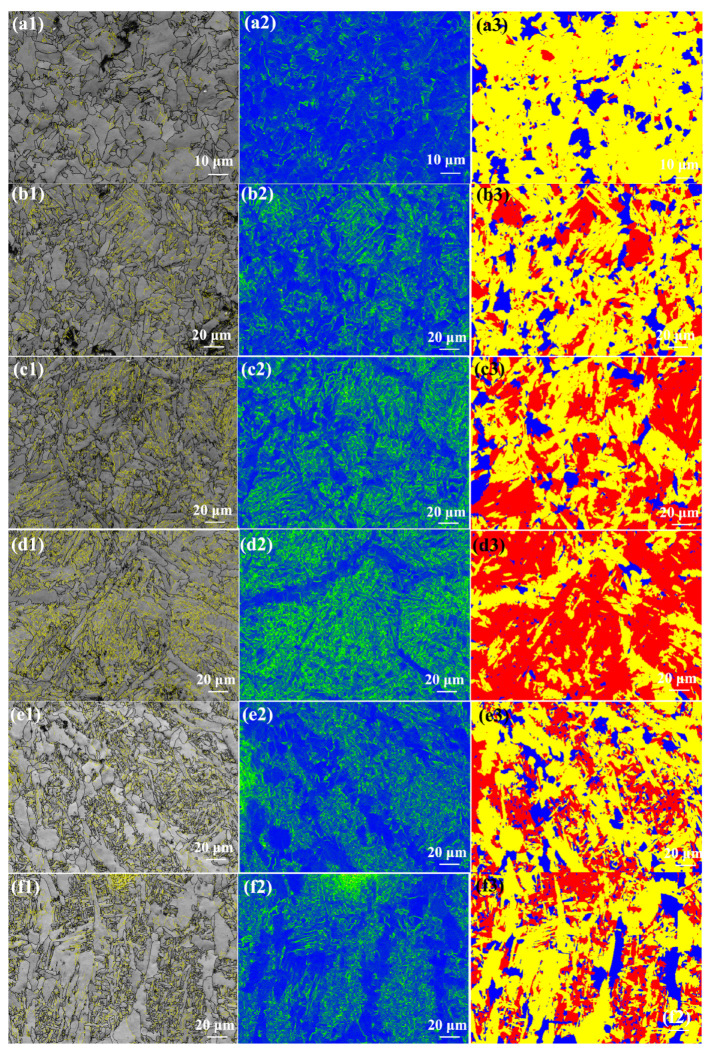
EBSD analysis of the as-welded SMAW joints (**a1**–**a3**) SCHAZ, (**b1**–**b3**) ICHAZ, (**c1**–**c3**) FGHAZ, (**d1**–**d3**) CGHAZ, (**e1**–**e3**) FZ. (**f1**–**f3**) NZ; (**a1**–**f1**) grain boundary distribution, (**a2**–**f2**) KAM, and (**a3**–**f3**) recrystallisation maps.

**Figure 11:**
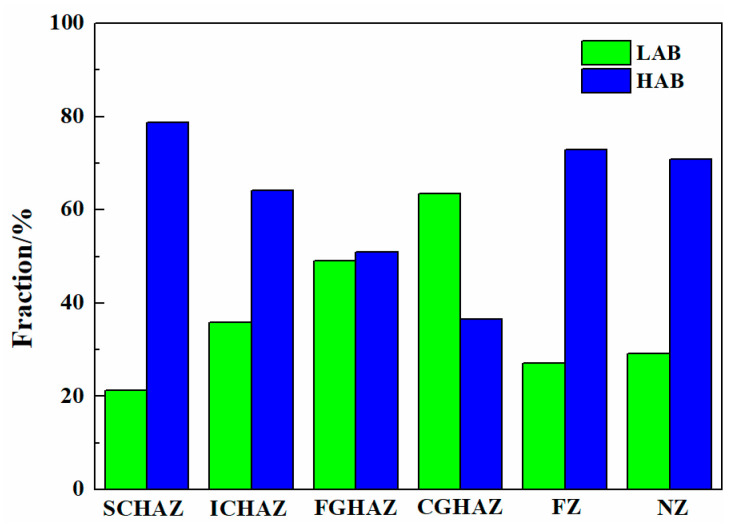
The grain boundary fraction in the as-welded SMAW joint.

Figure 10 shows the EBSD analysis of the as-welded SMAW joints. The black and yellow lines represent the high-angle boundaries (HABs, misorientation angle ≥ 15°) and low-angle boundaries (LABs, 2° ≤ misorientation angle < 15°), respectively. The grain boundary fraction in the as-welded SMAW joints is shown in Figure 11. The HAB fractions in the SCHAZ, ICHAZ, FGHAZ, CGHAZ, FZ, and NZ were 78.8%, 64.1%, 51%, 36.5%, 72.9%, and 70.9%, respectively. Clearly, in the HAZ, as the distance from the center of the NZ decreased, the fraction of HAB decreased because of grain growth. The fraction of HAB in the FZ and NZ increased relative to the HAZ, which was attributed to the presence of some fine ferrite. In addition, the KAM maps are used to characterize the local orientation gradient in grains caused by deformation. The high KAM value in grains implied the high dislocation density [19]. In the HAZ, the KAM value increased with the decrease in the distance from the NZ, while in the FZ and NZ, the KAM value decreased. In the HAZ, the fraction of bainite increased with the decrease in the distance from the center of NZ, which resulted in the increase in dislocation density and KAM. However, the presence of ferrite in the FZ and NZ decreased the KAM value. In the recrystallization maps, the yellow, red, and blue areas represent substructure, de-formation, and recrystallization microstructures, respectively. It was found that the FGHAZ and CGHAZ contained a large amount of deformed grains, while the FZ and NZ contained mainly substructures and recrystallized grains, which correspond to the low KAM values. This was mainly attributed to the slower post-welding cooling rate in the FZ and NZ, which was favorable for the recovery and annihilation of dislocations and substructures as well as the presence of some ferrite.

**Figure 12:**
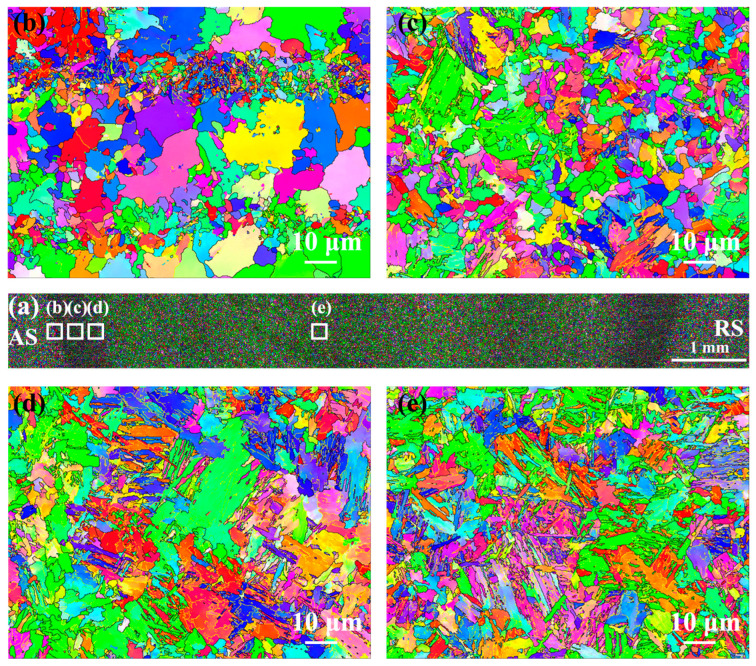
EBSD-IPF maps of the as-welded FSW joint (**a**) Macroscopic joint, (**b**) SCHAZ, (**c**) ICHAZ, (**d**) FGHAZ and (**e**) NZ.

Figure 12 shows EBSD-IPF maps of the as-welded FSW joint. In macroscopic joints, there was no as-cast microstructure in the NZ due to FSW belonging to the solid-phase welding technology (Figure 12a). There was no obvious difference in grain orientation distribution between HAZ and NZ (Figure 12b–e). Compared with SCHAZ, the average grain size in the ICHAZ, FGHAZ, and NZ was smaller, which may be due to transformation refinement. Compared with SMAW, the average grain size of FSW joint was obviously refined depending on low heat input.

**Figure 13:**
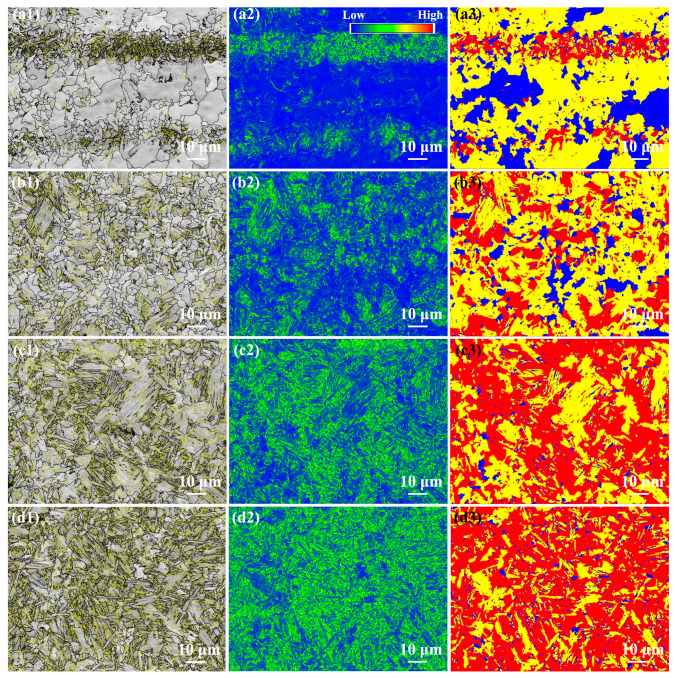
EBSD analysis of the as-welded FSW joint (**a1**–**a3**) SCHAZ, (**b1**–**b3**), ICHAZ, (**c1**–**c3**) FGHAZ, (**d1**–**d3**) NZ; (**a1**–**d1**) grain boundary distribution, (**a2**–**d2**) KAM and (**a3**–**d3**) recrystallization maps.

**Figure 14:**
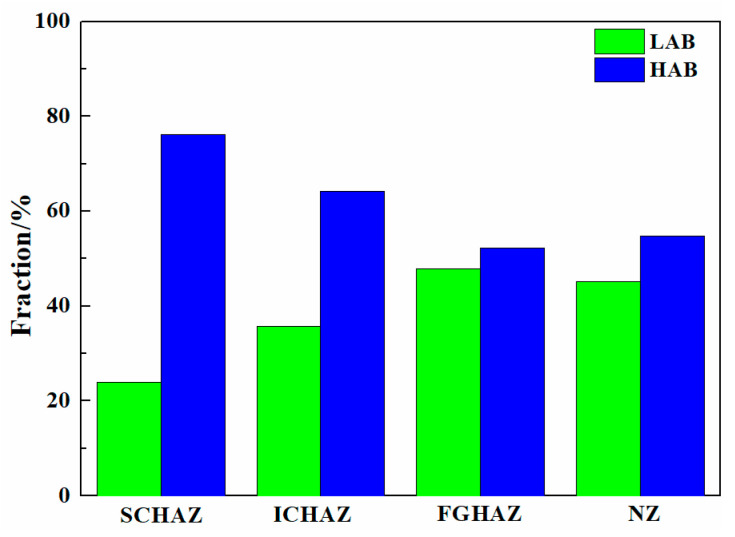
The grain boundary fraction in the as-welded FSW joint.

Figure 13 shows the grain boundary distribution, KAM and recrystallization maps of the as-welded FSW joint. The grain boundary fraction in the as-welded FSW joints is shown in Figure 14. The HAB fractions in the SCHAZ, ICHAZ, FGHAZ, and NZ were 76.1%, 64.2%, 52.2% and 54.8%, respectively. Obviously, with decreasing the distance from the NZ center, the fraction of LAB gradually increases, while the fraction of LAB gradually decreased. In the recrystallization map, the yellow, red, and blue areas represent substructure, deformation, and recrystallization microstructures, respectively. The variation in the KAM value is consistent with fraction of LAB, and there are a lot of deformed structures in the FGHAZ and NZ. Compared with FGHAZ, the number of deformed microstructures in the NZ increased obviously. During FSW, the NZ is not only affected by thermal action but also by plastic deformation, which leads to the accumulation of many dislocations in the post-welding microstructure. At the same time, the existence of martensite also leads to the increase in the KAM value and the proportion of LAB. Compared with the NZ of the SMAW joint, the decrease in the HAB fraction of NZ in FSW joint was mainly due to the introduction of high density of dislocations into NZ.

**Figure 15:**
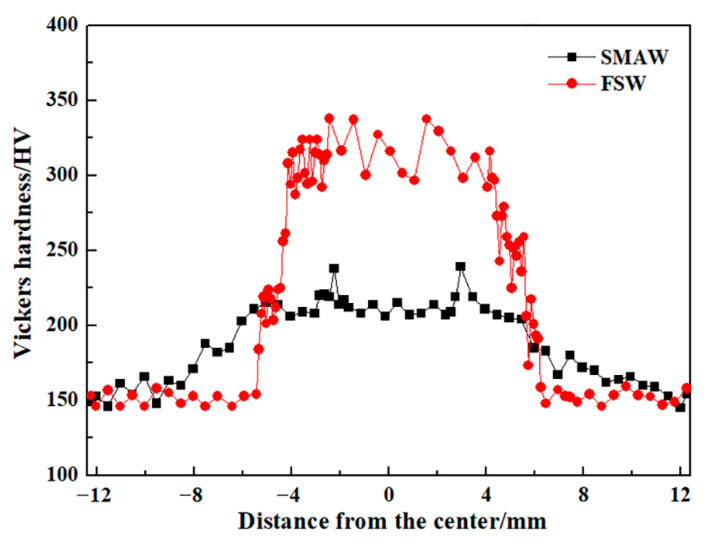
Vickers hardness distribution profile of the as-welded SMAW and FSW joints.

Figure 15 shows the Vickers hardness distribution of the as-welded SMAW and FSW joints. The BM exhibited the lowest hardness of approximately 150 HV corresponding to the soft ferrite and pearlite. Compared with BM, the hardness value of the HAZ and NZ increased significantly, irrespective of the welding process. In HAZ of SMAW joints, the hardness value increased with decreasing the distance from the center of the NZ, which was closely related to the content of lath bainite, while in the FZ and NZ, the hardness value decreased due to the appearance of soft ferrite. As for FSW joints, with decreasing the distance from the center of NZ, increasing hardness value depends on increasing content of bainite and martensite. In contrast, the hardness of the FSW joints was higher than that of SMAW joints, especially NZ, which corresponded to the existence of martensite, high density of dislocation, and fine grains. There are obvious soft zones in the resistance spot welded joints of medium Mn steel, which was attributed to that the long duration below the A_c1_ temperature resulted in the annealing softening of martensite [20]. However, in this work, the soft zones were not present in the FSW and SMAW joints.

**Figure 16:**
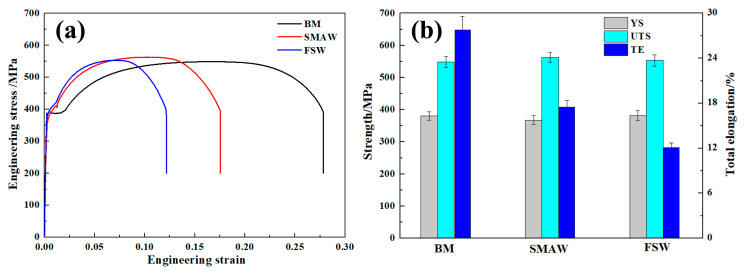
Tensile properties of the BM and as-welded SMAW and FSW joints. (**a**) engineering stress-strain curves, (**b**) tensile data.

Figure 16 shows tensile properties of the BM and as-welded SMAW and FSW joints. The fracture of SMAW and FSW joints occurred in the BM since the BM has the lowest hardness and gives priority to yield and fracture. The BM exhibited yield strength (YS) of 381 MPa, ultimate tensile strength of 549 MPa, and total elongation (TE) of 27.8%. In contrast, the SMAW joints exhibited a similar UTS of BM, while YS and TE of SMAW joints decreased to 368 MPa and 17.5%, respectively, due to a coarse microstructure. In the FSW joint, the YS and UTS, similar to that of BM, were obtained and depended on the low heat input. However, the TE in FSW joints decreased obviously to 12.1%. Compared to SMAW joints, the YS of FSW joints increased slightly due to the presence of martensite and high dislocation density and fine grains. However, the elongation of FSW joints decreased relative to SMAW joints. In the SA516 Gr.70 steel welded joint, based on the preferential yielding principle, the BM containing the lowest hardness preferentially yielded during tensile deformation, and the NZ with highest hardness was difficult to deform [21]. In this case, the width of NZ of the FSW joint was higher than that of the SMAW joint, thus resulting in a decrease in TE.

Table 2 lists the impact energies of the BM, HAZ, and NZ of as-welded SMAW and FSW joints at −46 °C. The impact energies in the HAZ and NZ of SMAW joint were 60 and 87 J/cm^2^, respectively, which were lower than that of the BM. In contrast, the impact energies in HAZ and NZ of the FSW joint were 80 and 89 J/cm^2^, respectively. Clearly, the impact energy of FSW joint was higher than that of SMAW joint, especially HAZ. It is well known that toughness is mainly affected by the grain size, microstructure, and grain boundary characteristics [13,22]. Generally, the soft phase and fine grain size were favored for improving toughness, and HABs can effectively change the crack propagation direction, thereby reducing the crack propagation rate and improving the toughness [12]. Although the microstructure of FSW joints contained some hard martensite relative to the SMAW joints, the finer grain size in FSW joints can help to improve toughness. Therefore, compared with SMAW joints, FSW joints exhibit high impact toughness.

Based on the above analysis, compared with SMAW joint, the toughness in joints of FSW SA516 Gr.70 steel was improved. However, the toughness and plasticity of the joint were not high due to the existence of hard phase bainite and martensite caused by the rapid post-welding cooling rate. Recently, some researchers have attempted to improve the toughness and plasticity of joints with hard phase microstructure. Cui et al. [23] performed FSW on medium Mn steel at different rotation rates to control the degree of martensite self-tempering and found that the degree of martensite tempering increased with the decrease in rotating speed. However, the degree of self-tempering was very limited due to the low M_s_ temperature of medium Mn steel. In addition, some scholars controlled the peak temperature location in the dual-phase region in FSW steel by using a very low rotation rate or introducing water cooling to obtain a soft ferrite microstructure in the NZ [24,25]. However, the lower peak temperature will aggravate the wear of stirring tools and increase the cost. Previously, Qi et al. [26] performed an intercritical annealing treatment on medium Mn steel joints with single martensite to improve the toughness and plasticity, and found that after annealing, dual-phase microstructures consisting of ferrite and austenite were obtained. During deformation, austenite excited an active transformation-induced plasticity (TRIP) effect, thus obtaining excellent toughness. In this case, post-welding annealing can effectively enhance the toughness and plasticity of FSW joints containing hard phase microstructure.

### 3.2. Effect of Annealing on the Microstructure and Mechanical Properties of the SMAW and FSW Joints

Given the heat treatment characteristics of SA516 Gr.70 steel, annealing was used in FSW and SMAW SA516 Gr.70 steel joints to improve the toughness and plasticity of the joints. During annealing, the microstructure experienced complex changes, which would have a significant impact on the mechanical properties of the joint. Therefore, the influence of annealing on the microstructure and mechanical properties of joints was investigated.

**Figure 17:**
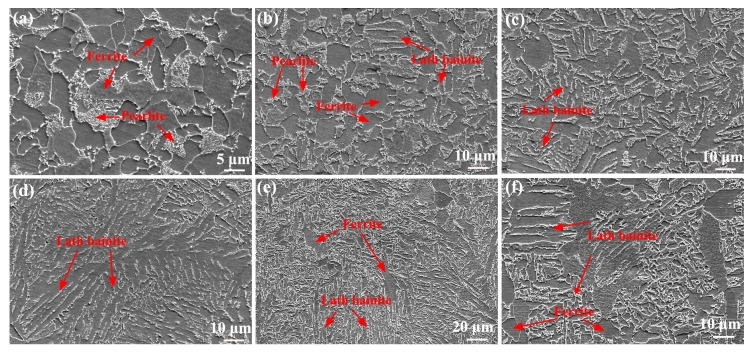
SEM images of as-annealed SMAW joint (**a**) SCHAZ, (**b**) ICHAZ, (**c**) FGHAZ, (**d**) CGHAZ, (**e**) FZ, (**f**) NZ.

Figure 17 shows SEM images of the as-annealed SMAW joints. The microstructure of SCHAZ was still pearlite and ferrite, and the carbon in pearlite was further diffused (Figure 17a). The ICHAZ was composed of ferrite, pearlite, and bainite, as shown by arrows in Figure 17b. The FGHAZ and CGHAZ contained mainly bainite, while the FZ and NZ consisted of ferrite and bainite. During annealing, carbon in pearlite or bainite diffused in the as-annealed SMAW joints and precipitated along the grain boundaries of ferrite matrix. Compared with the as-welded SMAW joint, the carbides appeared in ferrite matrix and grain size increased in the as-annealed SMAW joints after annealing.

**Figure 18:**
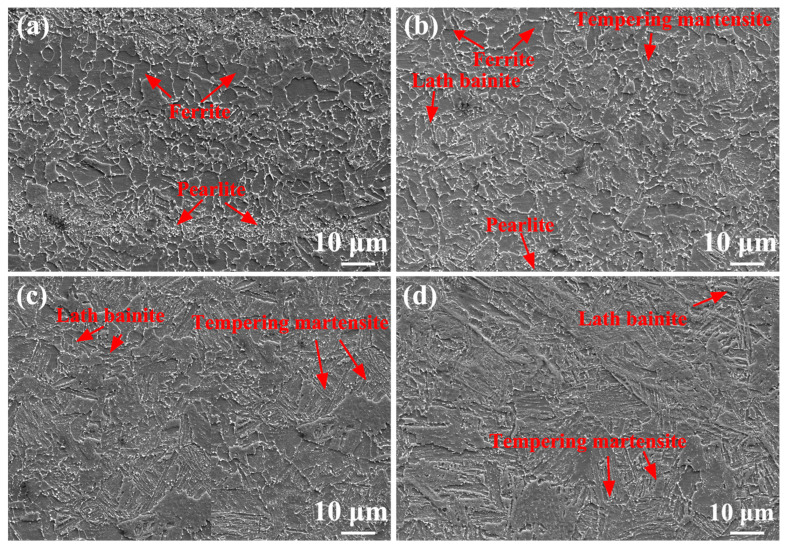
SEM images of as-annealed FSW joint (**a**) SCHAZ, (**b**) ICHAZ, (**c**) FGHAZ, (**d**) NZ.

Figure 18 shows SEM images of the as-annealed FSW joints. The microstructure of the SCHAZ was composed of ferrite and pearlite (Figure 18a), and carbon diffused in the pearlite during annealing. The microstructure of ICHAZ mainly consisted of ferrite, pearlite, bainite, and tempering martensite (Figure 18b). The microstructure of FGHAZ and NZ contained bainite and tempering martensite (Figure 18c,d). Compared with as-welded FSW joints, the carbon in pearlite, bainite, and martensite diffused to form carbides distributed along grain boundaries, and the grains grew in as-annealed FSW joints. Compared with as-annealed SMAW joint, the microstructure of as-annealed FSW joint was obviously refined.

**Figure 19:**
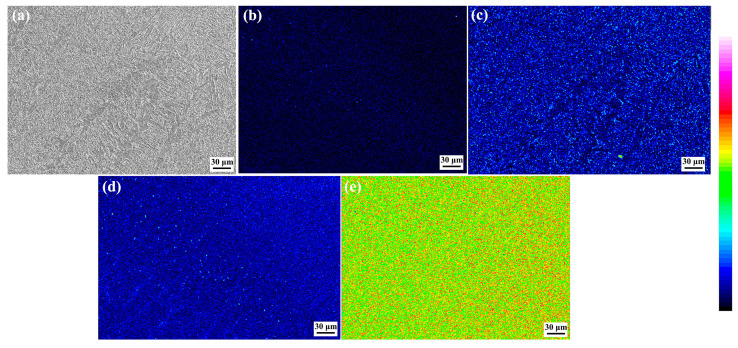
Element distribution maps in the as-annealed SMAW joint (**a**) SEM, (**b**) Si, (**c**) C, (**d**) Mn, (**e**) Fe.

Figure 19 shows the element distribution in the as-annealed SMAW joint. It can be found that the C, Mn, and Si elements in the FZ and HAZ are uniformly distributed without obvious segregation. Compared with as-welded FZ, the element distribution in the as-annealed FZ after annealing was uniform, indicating that post-welding annealing promoted the diffusion of elements, and finally obtaining the joint with uniform element distribution.

**Figure 20:**
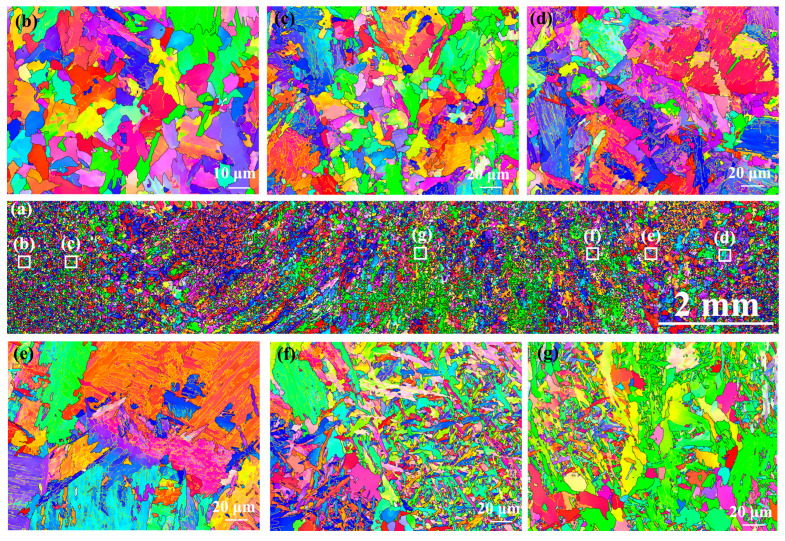
EBSD-IPF of as-annealed SMAW joint (**a**) Macroscopic joint, (**b**) SCHAZ, (**c**) ICHAZ, (**d**) FGHAZ, (**e**) CGHAZ, (**f**) FZ, (**g**) NZ.

Figure 20 shows the EBSD-IPF of the as-annealed SMAW joint. It can be found that the microstructure of NZ exhibited an as-cast morphology, which was obviously different from the HAZ. However, there was no obvious orientation distribution of grains in each sub-region of the welded joint. With decreasing the distance from the center of the NZ, the grain size first increased and then decreased. The average grain size of the FZ and NZ decreased relative to FGHAZ and CGHAZ because of the appearance of ferrite. Compared with the as-welded joint, the grain size in the as-annealed joints increased.

**Figure 21:**
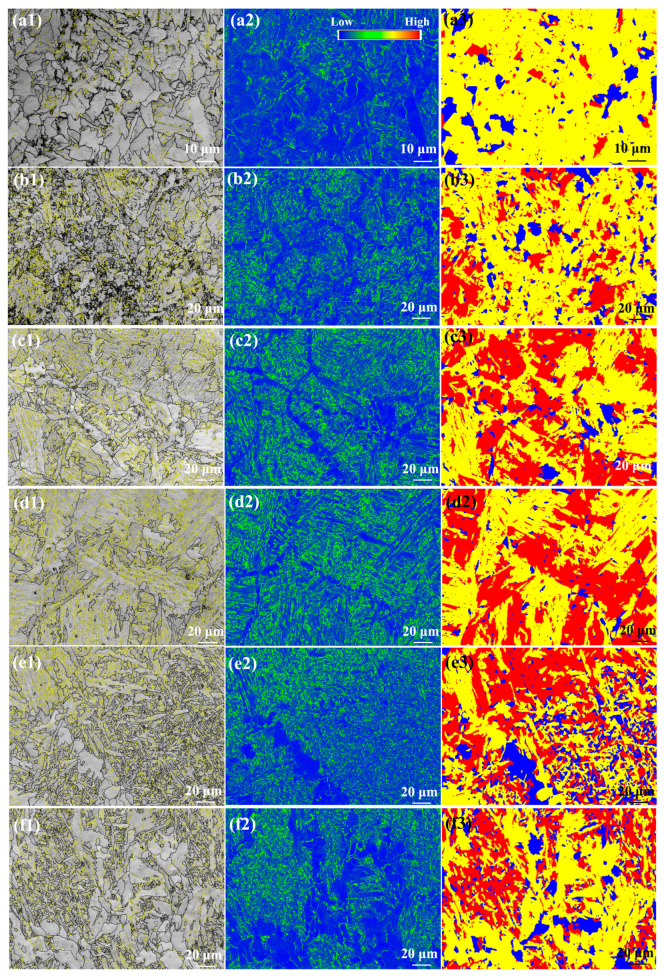
EBSD analysis of the as-annealed SMAW joint (**a1**–**a3**) SCHAZ, (**b1**–**b3**) ICHAZ, (**c1**–**c3**) FGHAZ, (**d1**–**d3**) CGHAZ, (**e1**–**e3**) FZ. (**f1**–**f3**) NZ; (**a1**–**f1**) grain boundary distribution, (**a2**–**f2**) KAM, and (**a3**–**f3**) recrystallization maps.

**Figure 22:**
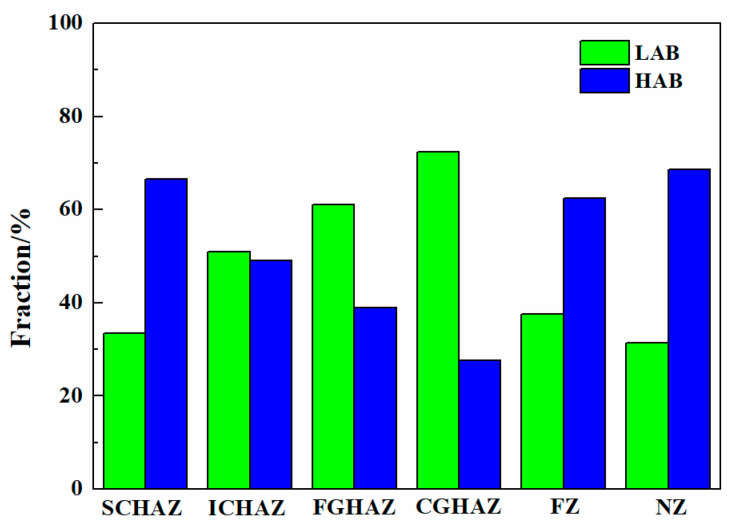
The grain boundary fraction in the as-annealed SMAW joint.

Figure 21 shows the EBSD analysis of the as-annealed SMAW joints. The grain boundary fraction in the as-annealed SMAW joints is shown in Figure 22. The HAB fractions in the SCHAZ, ICHAZ, FGHAZ, CGHAZ, FZ, and NZ were 66.6%, 49%, 38.9%, 27.6%, 62.4%, and 68.6%, respectively. Clearly, with decreasing the distance from the center of NZ, the fraction of HAB first decreased and then increased, while the KAM value and deformed microstructure content showed an opposite trend. The CGHAZ presented a low fraction of HAB and high KAM values, which corresponded to a high content deformation structure. Compared with as-welded, the KAM value and deformed microstructure of as-annealed joints were lower, especially for FGHAZ and CGHAZ, indicating that annealing can eliminate dislocation density and deformed microstructure.

**Figure 23:**
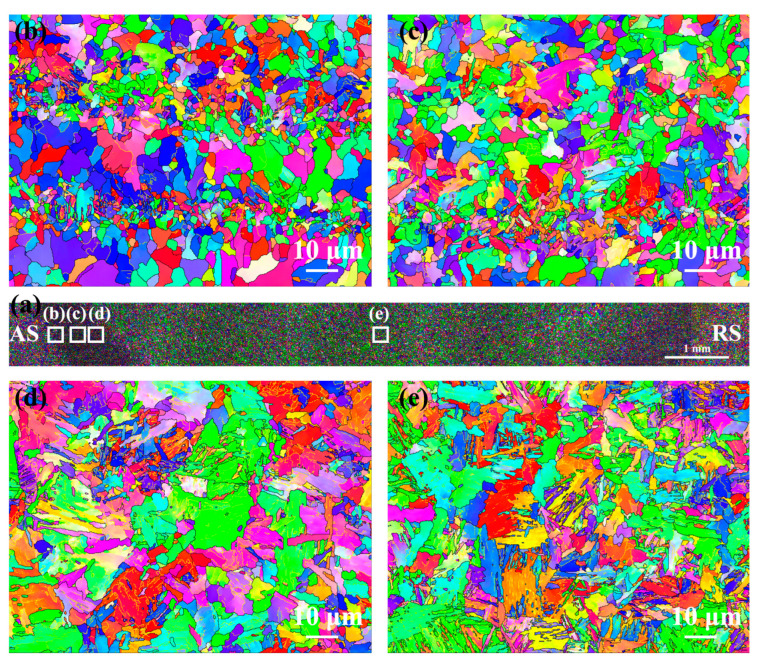
EBSD-IPF of as-annealed FSW joint (**a**) Macroscopic joint, (**b**) SCHAZ, (**c**) ICHAZ, (**d**) FGHAZ, (**e**) NZ.

Figure 23 shows the EBSD-IPF maps of as-annealed FSW joints. It can be found that the grain distribution in the HAZ and NZ presented equiaxed morphology, and there was no obvious difference in grain orientation distribution in each region. With decreasing distance from the center of the NZ, the grain size was obviously refined, which should be due to phase transformation refinement. Compared with as-welded FSW joints, the average grain sizes of the HAZ and NZ increased slightly after annealing, which were lower than those of as-annealed SMAW joints.

**Figure 24:**
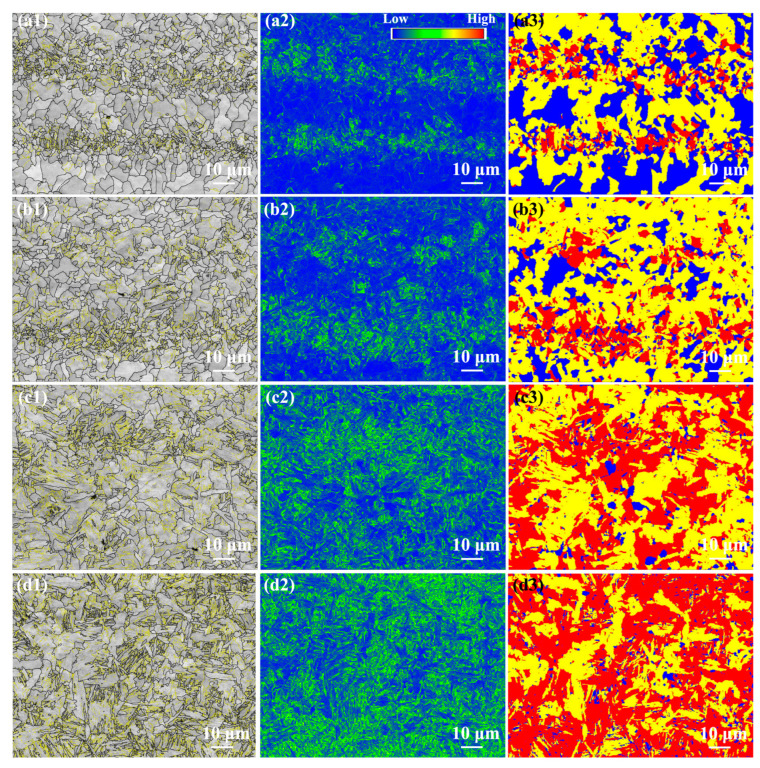
EBSD analysis of the as-annealed FSW joint (**a1**–**a3**) SCHAZ, (**b1**–**b3**), ICHAZ, (**c1**–**c3**) FGHAZ, (**d1**–**d3**) NZ; (**a1**–**d1**) grain boundary distribution, (**a2**–**d2**) KAM and (**a3**–**d3**) recrystallization maps.

**Figure 25:**
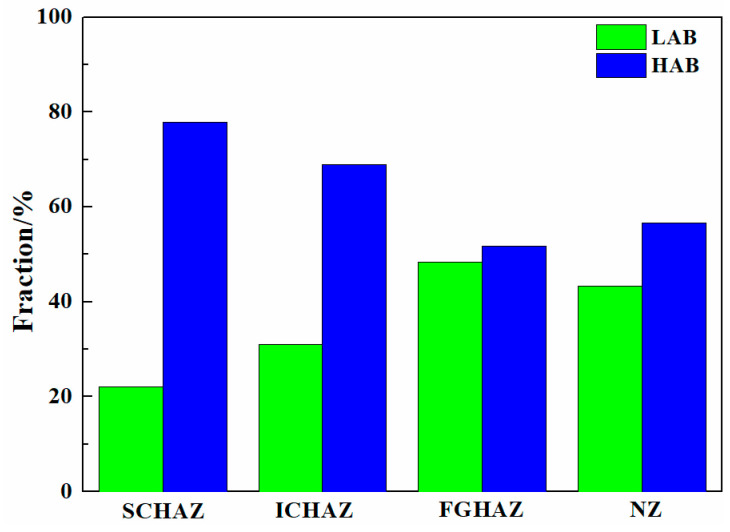
The grain boundary fraction in the as-annealed FSW joint.

Figure 24 shows the EBSD analysis in the as-annealed FSW joints. The grain boundary fractions in the as-annealed FSW joints are shown in Figure 25. The HAB fractions in the SCHAZ, ICHAZ, FGHAZ, and NZ were 77.9%, 69%, 51.7%, and 56.6%, respectively. With the decrease in the distance from the NZ center, the fraction of HAB first decreased and then increased, while the KAM value and deformed microstructure content increased gradually. Compared with ICHAZ and FGHAZ, the NZ exhibited a higher KAM value and a large amount of deformed microstructure, which was attributed to the plastic deformation of FSW during welding, resulting in a large number of dislocation substructures introduced into the NZ. Similarly, Lee et al. [27] pointed out that the high-density of dislocations was obtained in the NZ of FSW high Mn steel. Compared with as-welded FSW joints, after annealing, the KAM value and deformed microstructure content in the joints obviously decreased.

**Figure 26:**
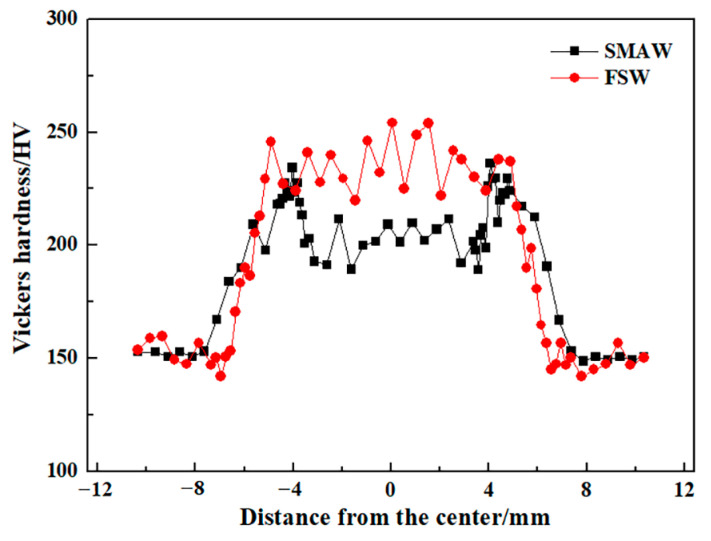
Vickers hardness distribution of the as-annealed SMAW and FSW joints.

Figure 26 shows the Vickers hardness distribution maps of the as-annealed SMAW and FSW joints. It was clear that the hardness value in the as-annealed FSW joint was higher than that of SMAW joint because of fine grain and many dislocations. However, compared with as-welded joints, the hardness values of FSW and SMAW joints after annealing decreased obviously, especially in FSW joints, which was attributed to that the appearance of tempering martensite and decrease in dislocation in bainite during annealing.

**Figure 27:**
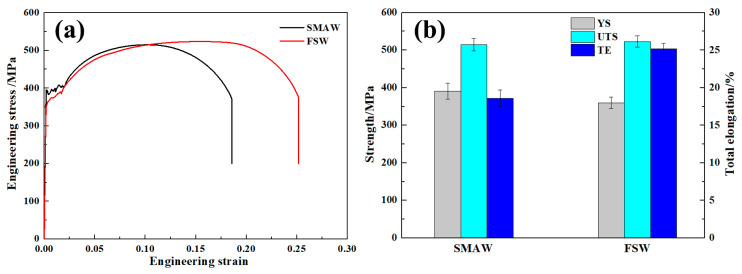
Tensile properties of the as-annealed SMAW and FSW joints (**a**) engineering stress-strain curves, (**b**) tensile data.

Figure 27 reveals the tensile properties of as-annealed SMAW and FSW joints. The YS, UTS, and TE of the as-annealed SMAW joint were 391 MPa, 515 MPa, and 18.6%, respectively. Compared with the as-welded SMAW joint, the as-annealed SMAW joint exhibited a similar TE, and the strength decreased. In contrast, the as-annealed FSW joint showed a UTS of 523 MPa and a high TE of 25.2%, which was much higher than that of the as-annealed SMAW joint. Obviously, compared with the as-welded FSW joint, the TE of as-annealed joint remarkably increased. After annealing, the difference between the maximum and minimum hardness values within the entire welded joint decreased because of the formation of a soft phase and a decrease in dislocation density. Therefore, compared to the as-welded joint, more zones in the as-annealed joint participated in the coordination deformation based on the preferential yielding principle, thereby improving the ductility of the joint. Similarly, Xue et al. [21] reported that a relatively small hardness difference in a pure copper joint was obtained by using FSW to join a pure copper under a water-cooling rate, producing a joint with excellent mechanical properties.

Table 3 lists the impact energy of the BM, HAZ, and NZ of as-annealed SMAW and FSW SA516 Gr.70 steel joints at −46 °C. The impact energy in the HAZ and NZ of the SMAW joint was 80 and 88 J/cm^2^, respectively. Compared with the as-welded SMAW joint, the impact energy of the as-annealed SMAW joint increased slightly. This was due to the hard phase bainite recovery during annealing. In contrast, impact energy in HAZ and NZ of FSW joint was 103 and 109 J/cm^2^, respectively, which was higher than that of the SMAW joint, especially HAZ that depended on the fine microstructure. Therefore, compared with as-welded FSW joints, the impact toughness of as-annealed FSW joints was remarkably improved, which was attributed to the fact that absence of hard martensite and bainite, and a decrease in the dislocation density during annealing.

Generally, the toughness and plasticity were mainly affected by the grain size and microstructure [13,22]. The soft phase and fine grain size are conducive to enhancing toughness and plasticity. In this work, after annealing, the microstructure in the joints of SMAW and FSW SA516 Gr.70 steel changed from hard phase to soft ferrite, and some carbides distributed along the grain boundary. Therefore, the main factor affecting the toughness and plasticity of as-annealed SMAW and FSW joints was grain size. Compared with as-annealed SMAW joints, the grain size of as-annealed FSW joints was obviously refined, which was attributed to the fact that FSW belonged to solid-phase welding, presenting low heat input and inhibiting grain growth.

In this work, SA516 Gr.70 steel joints exhibiting excellent toughness and plasticity were obtained by FSW and post-weld annealing, which effectively inhibited the appearance of coarsening and as-cast microstructure in fusion welded joints. In the following research, the welding parameters and annealing process will be further adjusted to further improve the toughness and plasticity of the joint. This systematic work provided theoretical guidance for improving joints with hard phase microstructure.

## 4. Conclusions

In this study, 4.5 mm thick SA516 Gr.70 steel was joined by SMAW and FSW and then was annealed to improve the toughness and plasticity. The microstructure and mechanical properties of as-welded and as-annealed joints were investigated in detail. The results are summarized as follows:

(1) In the SMAW joint, the HAZ consisted of ferrite, lath bainite, and pearlite; while with decreasing distance from the NZ, the ferrite and pearlite content decreased, the amount of bainite increased, and grain size increased markedly. For FSW joints, fine ferrite, pearlite, bainite, and martensite were obtained in the HAZ, and the ferrite and pearlite content decreased, and the amount of bainite and martensite increased as the distance from the NZ decreased.
(2) The Charpy energy of HAZ in FSW joint is 80 J/cm^2^, which is higher than that of HAZ in the SMAW joint (60 J/cm^2^) due to the microstructure refinement. In addition, the TE of the SMAW joint was 17.5%, which was higher than that of the FSW joint (12.1%), caused by wider NZ with high hardness.
(3) During annealing, carbon in pearlite or bainite diffused and precipitated along grain boundaries of the ferrite matrix in the as-annealed SMAW joints. With respect to the as-annealed FSW joints, tempering martensite, a decrease in dislocation density and carbides precipitating along grain boundaries was found.
(4) The toughness of HAZ of the as-annealed SMAW and FSW joints were 80 and 103 J/cm^2^, and TE of the as-annealed SMAW and FSW joints were 18.6% and 25.2%, respectively. Finally, the as-annealed FSW joints containing excellent toughness and ductility were obtained. The abovementioned excellent mechanical properties were primarily attributed to the appearance of tempering martensite, decrease in dislocation density, and fine grain.

## Figures and Tables

**Table 1:** Chemical compositions of SA516 Gr.70.

Component	C	Si	Mn	P	S	Fe
Content (wt.%)	0.16	0.32	1.47	0.016	0.0024	Bal.

**Table 2:** Impact energy of the as-welded joint at −46 °C (J/cm^2^).

V-Shaped Notch Locations	BM	HAZ	NZ
SMAW	115 ± 4	60 ± 4	87 ± 5
FSW	115 ± 5	80 ± 3	89 ± 4

**Table 3:** The impact energy of the as-annealed joints at −46 °C (J/cm^2^).

V-Shaped Notch Locations	HAZ	NZ
SMAW	80 ± 3	88 ± 4
FSW	103 ± 4	109 ± 5

## Data Availability

Data are contained within the article.
